# Adolescent metabolic syndrome and its components associations with incidence of type 2 diabetes in early adulthood: Tehran lipid and glucose study

**DOI:** 10.1186/s13098-020-00608-1

**Published:** 2021-01-02

**Authors:** Golaleh Asghari, Mitra Hasheminia, Abolfazl Heidari, Parvin Mirmiran, Kamran Guity, Mohammad Karim Shahrzad, Fereidoun Azizi, Farzad Hadaegh

**Affiliations:** 1 Nutrition and Endocrine Research Center, Research Institute for Endocrine Sciences, Shahid Beheshti University of Medical Sciences, Tehran, Iran; 2 Prevention of Metabolic Disorders Research Center, Research Institute for Endocrine Sciences, Shahid Beheshti University of Medical Sciences, Tehran, Iran; 3 Cellular and Molecular Endocrine Research Center, Research Institute for Endocrine Sciences, Shahid Beheshti University of Medical Sciences, Tehran, Iran; 4 Internal Medicine and Endocrinology, Shohada Tajrish Medical Center, Shahid Beheshti University of Medical Sciences, Tehran, Iran; 5 Endocrine Research Center, Research Institute for Endocrine Sciences, Shahid Beheshti University of Medical Sciences, Tehran, Iran

**Keywords:** Adolescent, Metabolic syndrome, Type 2 diabetes, Adulthood

## Abstract

**Aim:**

To investigate the association of youth metabolic syndrome (MetS) and its components, individually and in combination with early adulthood incident type 2 diabetes (T2DM).

**Methods:**

A total of 2798 adolescents aged 11–19 years enrolled in the study. At baseline, MetS, its components including blood pressure (BP), waist circumference (WC), triglycerides (TGs), fasting plasma glucose, and low HDL-C, and different combinations of MetS components were defined. After a mean 11.3 years of follow-up, T2DM was determined. Multivariable Cox proportional hazard regression analysis adjusted for age, sex, family history of T2DM, and adult BMI was used for data analysis. The hazard ratio (HR) and 95% confidence interval (CI) were reported.

**Results:**

During the follow-up, 44 incidents T2DM were developed. Among different individual components, only high WC [HR = 2.63, 95% CI (1.39–4.97)] and high TGs [HR = 1.82, 95% CI (1.00–3.34)] remained as significant predictors only in the age and sex adjusted model. Regarding combinations of MetS components, ‘high TGs and high WC’ [HR = 2.70, 95% CI (1.27–5.77)], ‘high BP and high WC’ [HR = 2.52, 95% CI (1.00–6.33)], ‘high TGs and high BP’ [HR = 2.27, 95% CI (1.02–5.05)] as well as MetS per se [HR = 2.82, 95% CI (1.41–5.64)] had a significant relationship with incident T2DM in the multivariable adjusted model. Among different confounders, being female and having family history of T2DM were consistently associated with higher risk of T2DM, in different combinations of MetS components.

**Conclusions:**

Adolescence MetS and some combinations of MetS components predicted early adulthood T2DM. Thus, adolescents, particularly female ones, with combinations of MetS components as well as those with family history of T2DM could be targeted for lifestyle intervention.

## Introduction

Metabolic syndrome (MetS) is considered as clustering of metabolic disorders including elevated blood pressure, abdominal obesity, hyperglycemia, and dyslipidemia [1] and rising trends of excess weight and MetS in adolescents is an alarming global concern [2]. A national study in Iran has reported that almost 5% of children and adolescents aged 7–18 years both in rural and urban areas had MetS [3]. The association of adult MetS and increased risk of non-communicable chronic diseases, viz. type 2 diabetes mellitus (T2DM) is well established [4, 5]. Diabetes prevalence increased from 4.3% in 1980 to 9.0% in 2014 in men, and from 5.0 to 7.9% in women [6]. In Iran, the estimated the national prevalence of diabetes in 2011 was 11.4%, which increased by 35% of reported values in 2005 among adult population [7].

Some studies suggest that pediatric metabolic abnormalities predict adult MetS [8, 9] and only a limited number of studies have investigated the association between MetS in youth and risk of future T2DM in adults [10–14]. Furthermore, contribution of each adolescent MetS components to risk of adult T2DM has been rarely considered [11, 13]. Magnussen et al. [13] in a study indicated that persistent MetS versus spontaneous resolution from MetS in youth increased risk of T2DM by 30–78% in adults. Furthermore, adolescents with MetS were at 2–3 times higher risk of having T2DM in adulthood compared with those free of MetS in childhood [11]. Although limited studies with different definitions of MetS, mostly without information on waist circumference (WC) investigating which definition could be a better predictor of incidence of chronic disease in adults, indicated that generally MetS among youth cannot be a better predictor than high BMI or overweight and obesity for prediction of adult outcomes [11]. There has not been an accurate and comprehensive examination about which combination of components can be more important in the development of adult T2DM.

Considering the growing prevalence of MetS in youth, and also T2DM incident in adulthood, as well as, the current lack of data from long-term cohort research on this issue, it seems vital that the association of youth MetS and its components with adult T2DM be investigated in large-scale longitudinal studies. The present study aimed to determine the association, in youth with metabolic disorders in the framework of MetS and its components in prediction of T2DM incidence in adults. Furthermore, we hypothesized that certain combinations of MetS components during adolescence may be more related with risk of T2DM in adulthood.

## Materials and methods

### Study design

This prospective study was conducted within the framework of the Tehran lipid and glucose study (TLGS), a large scale community-based program for monitoring the trend of non-communicable disease risk factors and developing a healthy lifestyle to reduce these risk factors. Baseline data were collected from 15,005 participants, aged ≥ 3 years, from District 13 of Tehran, under coverage of three medical health centers. The participants were followed every 3 years and all anthropometric, demographic, lifestyle, biochemical and clinical data were collected in each visit. The baseline survey was a cross-sectional study conducted from 1999 to 2001, and surveys 2 (2002–2005), 3 (2006–2008), 4 (2009–2011), and 5 (2012–2015) were prospective follow-up surveys.

**Figure 1:**
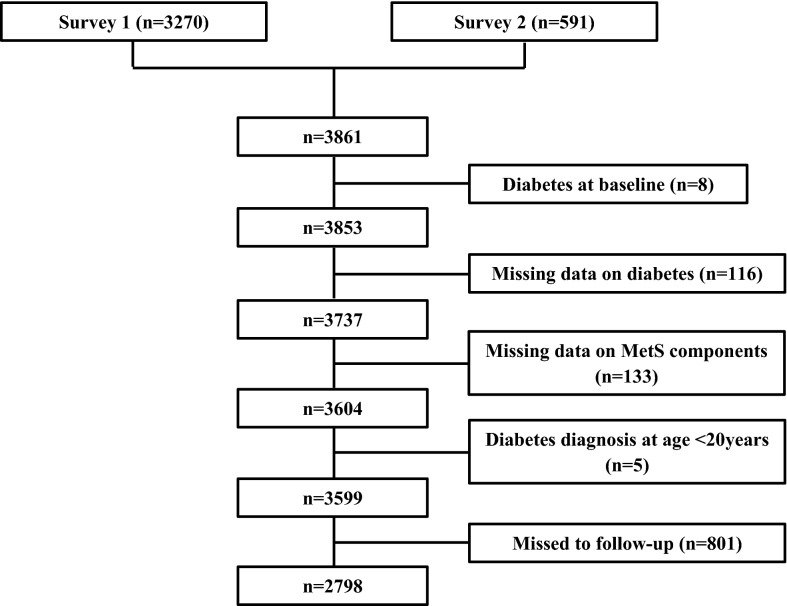
Fig. 1 Flow chart of the Tehran lipid and glucose study participants. *MetS* metabolic syndrome

From among 15,005 subjects recruited at baseline examination of the TLGS, 3861 adolescents aged 11–19 years were included from survey 1 (n = 3270) and 2 (n = 591). Participants with missing anthropometrical and biomechanical data (n = 254), diabetes at baseline (n = 8) and without any follow-up after recruitment (n = 801) were excluded from the analyses, therefore the final analysis was conducted in 2798 participants (response rate: 72.6%, Fig. 1).

### Measurements

Body mass (kg) was measured in minimal clothing without shoes to the nearest 0.01 kg, using a digital weighing scale (Seca 707, Hamburg, Germany). Height was measured, using a tape meter to the nearest 0.1 cm in a standing position, without shoes, and with shoulders in a normal alignment. Waist circumference (WC) was measured at the umbilical level over light clothing, using a tape meter nearest 0.1 cm over the skin, without any pressure to body surface. Body mass index (BMI) was calculated as body mass in kilograms divided by height in square meters.

After 12–14 h overnight fasting, a blood sample was taken between 07:00 and 09:00, at baseline and follow up. All blood analyses were done at the TLGS research laboratory on the day of blood collection. Fasting plasma glucose (FPG) at baseline and follow-up phases and 2‐h post‐challenge plasma glucose (2‐h PCPG) at follow-up phase were measured using an enzymatic colorimetric method with glucose oxidase (inter- and intra‐assay coefficients of variation at baseline and follow‐up phases were both less than 2.3%). High‐density lipoprotein cholesterol (HDL‐C) was measured after precipitation of the apolipoprotein B‐containing lipoproteins with phosphotungstic acid and serum triglycerides (TGs) were assayed using an enzymatic colorimetric method with glycerol phosphate oxidase. Both inter‐ and intra‐assay coefficients of variation were below 3 and 2.1% for HDL‐C and TGs, respectively, in all baseline and follow‐up assays. Analyses were performed using Pars Azmon kits (Pars Azmon Inc., Tehran, Iran) and a Selectra 2 auto‐analyzer (Vital Scientific, Spankeren, Netherlands).

### Definitions of MetS and T2DM

Because no universally accepted definition of the MetS exists for adolescents, the definition proposed by Cook et al. [15] was used. The MetS was defined as 3 or more of the following: Fasting TGs ≥ 110 mg/dl; HDL-C < 40 mg/dl; WC ≥ 90th percentile for age and sex, according to national reference curves [16]; systolic blood pressure and/or diastolic blood pressure ≥ 90th percentile for sex, age, and height, based on the recommended cut-off points of the National Heart, Lung, and Blood Institute [17] and for fasting blood glucose ≥ 100 mg/dl, according to recent recommendations of the American Diabetes Association [18].

Type 2 diabetes was defined according to criteria of the American Diabetes Association criteria. Participants aged ≥ 20 years old, who met at least one of these criteria were considered as having T2DM: FPG ≥ 126 mg/dl, 2-h PCPG ≥ 200 mg/dl or taking anti‐diabetic medication [19]. However, for participants aged < 20 years, T2DM was defined as FPG ≥ 126 mg/dl or taking anti‐diabetic medication.

### Statistical analysis

Mean ± standard deviation (SD) for continuous and frequency (%) for categorical variables were used to show baseline characteristics of participants. Triglycerides had skewed distribution and were shown as median (25–75 interquartile range). Comparison of baseline characteristics between participants with and without incident T2DM as well as respondent and non-respondent (those with missing data and those who did not have any follow-up) were done using Student’s t-test for continuous variables, Chi-square test for categorical variables and Mann-Whitney test for skewed variables. Cox proportional hazard regression analysis was used to assess the association of MetS components with incident T2DM.

In the current study, the survival time, being either the time between entrance the study and incident T2DM or the time between entrance the study and censor (i.e. participants for whom information on time to event is not available due to loss to follow-up or non-occurrence of incident T2DM before the study end). Because the exact time of onset of diabetes is not known in the TLGS, the event time is set as the half-time survival between when diabetes was first diagnosed and the last visit before diagnosis. Univariable Cox analysis was performed for each potential risk factor including age, sex, adulthood BMI, and family history of T2DM. Covariates with a P-value < 0.2 in the initial univariable analysis were selected to enter the multivariable model. The first model was adjusted for age, sex and the second one was further adjusted for adulthood BMI and family history of T2DM. The proportional hazards assumption in the Cox model was assessed with the Schoenfild residual test and all proportionality assumptions were appropriate. The incidence density rate of T2DM was calculated as the number of new (incident) cases during study follow up divided by the person-time-at risk throughout the observation period and reported per 10,000 person years of follow up for MetS, its components, and combinations of MetS components. Statistical analyses were performed using SPSS for windows version 20 and STATA version 12, with P-values ≤ 0.05 being considered statistically significant.

## Results

In the current study, 1279 male 1519 female adolescents with mean (± SD) age of 14.3 (2.8) and 14.7 (2.8) years were included, respectively. The mean age of participants at the last follow-up was 26.0 (4.4) years. Comparing baseline characteristics between respondents and non-respondents, WC and sex were different (P < 0.05); however, the difference was not clinically important, hence, our cohort did not reflect a selection bias at the time of follow-up (Table 1).

**Table 1:** Mean differences for baseline characteristics between study respondents and non-respondents in the Tehran lipid and glucose study

	Respondents	Non-respondents	Mean difference	*P*-value
Age (years)	14.5 ± 2.8	14.7 ± 2.8	0.16	0.125
Body mass index (kg /m^2^)	20.5 ± 4.2	20.8 ± 4.7	0.29	0.088
WC (cm)	70.3 ± 10.8	71.5 ± 11.8	1.17	0.007
SBP (mmHg)	105.3 ± 11.8	105.5 ± 11.9	0.49	0.261
DBP (mmHg)	70.8 ± 9.4	71.1 ± 9.0	0.28	0.426
HDL-C (mg/dl)	42.9 ± 10.1	42.6 ± 10.4	− 0.24	0.540
TGs (mg⁄dl)	96 (71–132)	92 (69–128)	− 0.55	0.806
FPG (mg⁄dl)	88.0 ± 7.8	87.6 ± 8.0	− 0.47	0.116
Female (%)	54.3	50.2		0.025

Respondents were defined as those with full data and have at least one follow-up and non-respondents were defined as those with missing data and those who did not have any follow-up

Data are given as the mean ± SD or median (IQ 25–75) unless otherwise indicated

As shown in Table 2, there were no significant differences between participants with and without incident T2DM in SBP, DBP, HDL-C, and FPG (all P-values > 0.05), whereas, participants with onset of T2DM had higher BMI, WC, and TGs (*P* < 0.01). Furthermore, those with incident T2DM were more likely to be female, had higher prevalence of high WC (31.8% vs. 16.8%, *P* = 0.006), high TGs (50.0% vs. 36.6%, *P* = 0.067), and prevalence of MetS (29.5% vs. 11.9%, *P* < 0.001).

**Table 2:** Baseline characteristics between adolescents with incident type 2 diabetes and those without incident type 2 diabetes in the Tehran lipid and glucose study

	With incident type 2 diabetes (n = 44)	Without incident type 2 diabetes (n = 2754)	*P*-value	Total (n = 2798)
Continuous variables				
Age (years)	15.2 ± 2.6	14.5 ± 2.8	0.110	14.5 ± 2.8
Body mass index (kg /m^2^)	22.2 ± 4.7	20.5 ± 4.2	0.005	20.5 ± 4.2
WC (cm)	74.6 ± 10.6	70.3 ± 10.8	0.009	70.3 ± 10.8
SBP (mmHg)	105.8 ± 14.0	105.0 ± 11.7	0.649	105.0 ± 11.8
DBP (mmHg)	72.6 ± 9.6	70.8 ± 9.4	0.200	70.8 ± 9.4
HDL-C (mg/dl)	42.8 ± 10.2	42.9 ± 10.1	0.950	42.9 ± 10.1
TGs (mg⁄dl)	117 (86–152)	95 (71–131)	0.015	96 (71–132)
FPG (mg⁄dl)	89.6 ± 9.0	88.0 ± 7.8	0.194	88.0 ± 7.8
Categorical variables				
Female (%)	81.8	53.8	< 0.001	54.3
High WC (%)	31.8	16.3	0.006	16.5
High BP (%)	22.7	16.3	0.254	16.4
Low HDL-C (%)	52.3	46.2	0.422	46.3
High TGs (%)	50.0	36.6	0.067	36.8
High FPG (%)	9.1	7.1	0.607	7.1
Metabolic syndrome (%)	29.5	11.9	< 0.001	12.1

Data are given as the mean ± SD or median (IQ 25–75) unless otherwise indicated

Metabolic syndrome was defined as 3 or more of the following: TGs ≥ 110 mg/dl; HDL-C < 40 mg/dl; FPG ≥ 100 mg/dl; WC ≥ 90th percentile for age and sex, according to national reference curves [16]; and SBP and/or DBP ≥ 90th percentile for sex, age, and height, according to the National Heart, Lung, and Blood Institute’s recommended cut-off points [17]

*WC* waist circumference, *SBP* systolic blood pressure, *DBP* diastolic blood pressure, *HDL-C* high-density lipoprotein cholesterol, *TGs* triglycerides, *FPG* fasting plasma glucose

During the median follow-up of 11.3 years, 44 (girls = 38) new cases of T2DM were developed with a corresponding incidence rate of 13.89 per 10,000 person-years.

Table 3 shows the association between adolescent MetS and its components with the risk of early adulthood T2DM incident. Focusing on MetS components, in the age and sex adjusted model, high WC and high TGs had higher risk of T2DM. However, after further adjustment for adulthood BMI and family history of T2DM (model 2), none of the MetS components individually remained as a predictor. Regarding different combinations of MetS components, significant HR (95% CI) in the fully adjusted model for incidence of T2DM was 2.70 (1.27–5.77) for ‘high TGs and WC’—2.52 (1.00–6.33) for ‘high BP and WC’—2.27 (1.02–5.05) for ‘high TGs and BP’—and 2.82 (1.41–5.64) for MetS. However ‘low HDL-C and high WC’, ‘high TGs and low HDL-C’ and ‘low HDL-C and high BP’ were predictors of T2DM only in the first model.

**Table 3:** Hazard ratio and 95% confidence interval for incidence of type 2 diabetes in early adulthood based on metabolic syndrome and its components, 1999–2015 (n = 2798)

	n	Incidence rate (per 10,000 person-years)	Model 1	Model 2
Components				
High WC	462	27.95	2.63 (1.39–4.97)*	1.92 (0.93–3.97)
High BP	456	18.74	1.58 (0.77–3.22)	1.37 (0.66–2.81)
Low HDL-C	1295	15.68	1.20 (0.66–2.18)	1.11 (0.61–2.01)
High TGs	1030	18.70	1.82 (1.00–3.34)*	1.68 (0.92–3.09)
High FPG	199	17.19	1.51 (0.53–4.26)	1.49 (0.53–4.22)
Dual combinations				
High TGs and high WC	285	34.79	3.56 (1.78–7.10)*	2.70 (1.27–5.77)*
High FPG and high WC	39	21.53	1.70 (0.23–12.42)	1.37 (0.19–10.09)
Low HDL-C and high WC	269	27.12	2.42 (1.12–5.23)*	1.66 (0.71–3.85)
High BP and high WC	126	43.37	3.84 (1.62–9.13)*	2.52 (1.00–6.33)*
High TGs and high FPG	87	19.58	1.58 (0.38–6.61)	1.50 (0.36–6.28)
High TGs and low HDL-C	621	21.14	1.86 (0.99–3.49)*	1.68 (0.89–3.17)
High TGs and high BP	215	31.38	2.83 (1.29–6.16)*	2.27 (1.02–5.05)*
High FPG and low HDL-C	92	27.54	2.41 (0.74–7.83)	2.08 (0.64–6.80)
High FPG and high BP	36	42.33	3.79 (0.91–15.76)	3.35 (0.80-14.08)
Low HDL-C and high BP	190	31.47	2.67 (1.18–6.01)*	2.15 (0.94–4.90)
Metabolic syndrome	338	33.78	3.55 (1.84–6.87)*	2.82 (1.41–5.64)*

Incidence rate, number of incident type 2 diabetes cases divided by person-year follow up

Model 1 is adjusted for age and sex; model 2 is further adjusted for adulthood body mass index and family history of diabetes

*WC* waist circumference, *SBP* systolic blood pressure, *DBP* diastolic blood pressure, *HDL-C* high-density lipoprotein cholesterol, *TGs* triglycerides, *FPG* fasting plasma glucose

*P-value ≤ 0.05

According to Table 4, for all significant predictor combinations of MetS components, being female and family history of T2DM were consistently associated with higher risk of T2DM. However, adulthood BMI was associated with higher risk of incident T2DM, which trend to be significant for ‘high BP and WC’ and ‘high TGs and BP’.

**Table 4:** Multivariable adjusted HR (95% CI) for confounders including age, sex, adulthood body mass index, and family history of diabetes of each significant combination of MetS components in predicting early adulthood type 2 diabetes (n = 2798)

	High TGs and WC	High BP and WC	High TGs and BP	MetS
Age	1.07 (0.96–1.20)	1.06 (0.95–1.18)	1.07 (0.95–1.19)	1.07 (0.96–1.20)
Female	4.56 (2.10–9.88)^*^	4.41 (2.04–9.54)^*^	4.45 (2.06–9.61)^*^	4.70 (2.17–10.21)^*^
Adulthood body mass index	1.04 (0.98–1.10)	1.05 (1.00–1.11)^†^	1.06 (1.00–1.12)^†^	1.05 (0.99–1.10)
Family history of diabetes	2.93 (1.55–5.54)^*^	2.87 (1.52–5.44) ^*^	2.90 (1.53–5.49)^*^	2.91 (1.54–5.51)^*^

*MetS* metabolic syndrome, *TGs* triglycerides, *WC* waist circumference, *BP* blood pressure

^*^P-value < 0.05

^†^P-value = 0.06

## Discussion

The present study demonstrated three key aspects regarding the impact of adolescent MetS and its components in prediction of early adulthood T2DM. First, although ‘high WC and high TGs’ were associated with incident T2DM, the risk significantly attenuated after further adjustment of adulthood BMI and family history of T2DM. Second, different combinations of MetS components, which included high TGs, high BP, and high WC as well as prevalence of MetS per se, were associated with more than 2-time higher risk of T2DM in the fully adjusted model. Third, among different confounders in our data analysis, being female and having family history of T2DM remained as powerful predictors for incident T2DM.

To the best of our knowledge, this is the first study in the Middle East North Africa (MENA) region to investigate whether adolescent MetS components could predict early adulthood T2DM, independent of adult BMI and family history of DM. Our findings have important implications because of the high burden of MetS and T2DM in the MENA region [20].

A comparison of our findings with other studies is difficult because of the differences in MetS definition, confounder selection, T2DM definition as an outcome, and participant age at recruitment. Most of the previous studies have been conducted in the North America and West Europe. For example, Morrison et al. [9] showed that children and adolescents aged 5–19 years with MetS were more likely to have T2DM 25 to 30 years later than their peers. In univariate models, childhood MetS, family history of diabetes, age at follow-up, and black race were associated with adult T2DM, although sex and change in BMI were not significant [9]. In addition, Magnussen et al. [11] showed that despite the instability of adolescent MetS over a 24-year period, it predicted T2DM in early to middle adulthood. Our data are also consistent with the National Growth and Health Study, which showed that both childhood high insulin and MetS independently predicted impaired fasting glucose and T2DM 14 years later [21]. In contrast, Koskinen et al. [12] found no association between adolescent obesity phenotypes (metabolic disturbances even in the presence of obesity) and adult T2DM. When comparing these studies, the important explanation may be the adjusting variables. Beside age and sex, we considered two other important confounders including adult BMI and family history of DM; the variables that less included in other similar studies. Adult BMI is an important confounding variable as it has been previously shown that childhood and adolescence obesity is associated with increased risk of adult MetS without adjustment of adult BMI; however, after adjustment for adult BMI, the association was ameliorated or inversed [22].

Among components of MetS in our study, high TGs and high WC associated early adulthood T2DM; however, after adjustment for family history of T2DM and adult BMI, the association was attenuated. Other individual components of MetS did not show any association with T2DM. Similarly, in the observational ‘Bogalusa Heart and Cardiovascular Risk in Young Finns’ studies, none of the MetS components predicted adult T2DM [11].

In addition to MetS and its individual components, some combinations of MetS components, which are common in high BP, high TGs, and high WC, may be have more prediction power than high FPG and low HDL-C for T2DM in the current study. It is important to note that in dual combinations, which did not include high TGs and high WC, we showed the effect size more than two, ranging from 2.41 (0.74–7.83) in model including high FPG and low HDL-C to 3.79 (0.91–15.76) in model including high FPG and high BP. However, despite the magnitude of HRs, because of the wide confidence intervals, these effect sizes were not stable. Therefore, we cannot firmly conclude that ‘high WC and high TGs’ combination had higher influence on incident T2DM than the other dual combinations, which did not include these parameters. Current evidence shows the role of hypertension, hypertriglyceridemia, low HDL-C, and obesity indices like high BMI and WC with insulin resistance [23–28]. Although, these factors have close biochemical relations, and generally overlap and coordinate together, only limited studies have investigated their interactions in the development of insulin resistance [29, 30].

Perhaps the most important finding from this study is that family history of T2DM predicts early adulthood T2DM as well as or better than MetS and its component combinations. Similarly, cohort studies of the Princeton Follow-up Study and the National Growth and Health Study, indicated parental history of T2DM to be an independent predictor of T2DM among children and adolescents [31, 32]. This finding has significant clinical relevance because during pediatric visits, family history of T2DM can easily be determined and allows the immediate identification of adolescents at increased risk who might benefit from therapeutic lifestyle intervention.

Another finding of this study was that in multivariate analysis, high FPG was associated with about 50% increased risk of incident T2DM, the value that did not reach to significant level, considering that high FPG was the least prevalent component of MetS in our population. This is in contrast to results of our previous study, which indicates that continuous FPG predicted adult T2DM [33]. The discrepancy of findings between dichotomous and continuous FPG for prediction of T2DM may due to definition of high FPG (FPG ≥ 100 mg/dl). Similarly, childhood glucose, as an explanatory variable along with lipids, blood pressure, obesity, and family history of T2DM did not change the model for development of T2DM [32], although high childhood glucose was an independent predictor of impaired fasting glucose and T2DM in both the Princeton Follow-up Study and the National Growth and Health Study [21, 31]. Therefore, it seems that to show the clinical utility of FPG component of MetS, individually or in combination, longer studies with higher number of incident case of T2DM is required. Furthermore, the non-significant finding of low HDL-C may be explained by the low variation of HDL-C in our study population, i.e. approximately 46.3% of participants had low HDL-C, with a mean of 42.9 ± 10.1 mg/dl.

Our study has noteworthy limitations. We did not obtain fasting serum insulin in adolescents as this variable gives more objective measures of insulin resistance. Furthermore, the low number of some combinations of MetS components (particularly those including high FPG) and low incident cases of T2DM lead to an unstable effect sizes as high as 3.35 (for ‘high FPG and high BP’) with wide CI. In addition, some possible confounders such as puberty stage, physical activity, dietary habits, and socioeconomic status were not taken into account. The main strengths of our study were that the parenteral history of T2DM was determined by FPG ≥ 126 mg/dl, and or 2-h post prandial plasma glucose ≥ 200 mg/dl and or drug consumption rather than self-reported T2DM. In addition, we used WC to define abdominal adiposity which is more reliable for metabolic adiposity in the context of defining MetS. This measurement was a major limitation for previous cohort studies in pediatrics [9, 11].

In conclusion, adolescent MetS and some dual combinations of MetS components including high WC, TGs, and BP, predicted early adulthood T2DM, independent of family history of T2DM and adulthood BMI. Family history of T2DM in all models was a strong predictor of T2DM. These findings have practical clinical value in assessment of adolescents, aged 11–19 years, since adolescents with high WC, TGs, and BP, and family history of T2DM could be targeted for lifestyle interventions, with special focus on adolescents with a positive family of T2DM.

## Data Availability

The datasets generated and/or analyzed during the current study are not publicly available due institution’s policy but are available from the corresponding author on reasonable request.
